# Greater Utilization of Neural-Circuits Related to Executive Functions is Associated with Better Reading: A Longitudinal fMRI Study Using the Verb Generation Task

**DOI:** 10.3389/fnhum.2014.00447

**Published:** 2014-06-20

**Authors:** Tzipi Horowitz-Kraus, Jennifer J. Vannest, Elveda Gozdas, Scott K. Holland

**Affiliations:** ^1^Cincinnati Children’s Research Foundation, Pediatric Neuroimaging Research Consortium, Cincinnati Children’s Hospital Medical Center, Cincinnati, OH, USA

**Keywords:** development, dual-networks model, executive functions, fMRI, reading, verb generation

## Abstract

**Introduction:** Reading is an acquired-developmental ability that relies on intact language and executive function skills. Verbal fluency tasks (such as verb generation) also engage language and executive function skills. Performance of such tasks matures with normal language development, and is independent of reading proficiency. In this longitudinal fMRI study, we aim to examine the association between maturation of neural-circuits supporting both executive functions and language (assessed using verb generation) with reading proficiency achieved in adolescence with a focus on left-lateralization typical for language proficiency.

**Methods:** Normalized fMRI data from the verb generation task was collected from 16 healthy children at ages 7, 11, and 17 years and was correlated with reading scores at 17 years of age. Lateralization indices were calculated in key language, reading, and executive function-related regions in all age groups.

**Results:** Typical development was associated with (i) increasingly left-lateralized patterns in language regions (ii) more profound left-lateralized activation for reading and executive function-related regions when correlating with reading scores, (iii) greater involvement of frontal and parietal regions (in older children), and of the anterior frontal cortex (in younger children).

**Conclusion:** We suggest that reading and verb generation share mutual neural-circuits during development with major reliance on regions related to executive functions and reading. The results are discussed in the context of the dual-networks architecture model.

## Introduction

The normal course of development involves continued improvement in higher-order cognitive abilities (e.g., executive functions), that originate in prefrontal cortex (PFC) (Diamond, 2002 for review). There is strong evidence supporting the continued anatomical and functional maturation of the PFC related to development of these skills (Sowell et al., 1999). The cognitive skills gathered under the umbrella term “Executive functions” include a wide range of conscious processes involved in monitoring and optimizing performance (Baddeley, 1986) such as error monitoring, selective attention to the relevant stimulus, inhibiting execution of undesired responses, working memory (WM) abilities (the ability to manipulate and maintain information), shifting, and fluency.

Fluency is part of the information processing component of executive functions and reflects the integrity of neural connections and the functional integration of frontal systems. Fluency can be evaluated by the speed, quantity, and quality of output (Anderson, 2002). Semantic verbal fluency ability is typically assessed either by semantic category fluency (generating members of a category) or by verb generation in response to auditory or visual nouns. Individuals performing a semantic category fluency task, exhibit activation of the left hemisphere regions related to language and articulation [inferior frontal gyrus (IFG), representing word retrieval; middle frontal gyrus (MFG) attributed to verbal WM, and supplementary motor area (SMA), which reflects attention and motor planning] (Paulesu et al., 1997; Hugdahl et al., 1999; Pihlajamaki et al., 2000; Gaillard et al., 2003) in both children and adults (Gaillard et al., 2003). The verb generation task also reflects semantic verbal fluency ability (Piatt et al., 1999). In this task, participants are presented with a noun, either auditorily or visually, and are asked to generate related verbs. Similar to semantic category fluency, the verb generation task has been found to activate several frontal regions [left and right IFG (BA 44, 45, 46, 47), and the left medial frontal gyrus (MFG) (BA 6, 8, 9)] as well as language regions in the brain [left and right medial temporal gyrus (BA 19/39, 21), right and left inferior temporal gyrus (ITG) (BA 19/37), and superior temporal gyrus (STG) (BA 22)] in participants aged 5–18 years of age (Holland et al., 2001, 2007; Karunanayaka et al., 2010, 2011). We have also documented developmental changes in lateralization of the language-network supporting the verb generation task in both a cross-sectional (Holland et al., 2007) and a longitudinal study (Szaflarski et al., 2006). Specifically, the network becomes increasingly left-lateralized with age. Karunanayaka examined developmental changes in neurocognitive modules underlying the verb generation task using Independent Component Analysis (Karunanayaka et al., 2011). Two primary neurocognitive modules were identified in that work: (1) word processing [auditory (BA 22), and then phonological processing (BA 45/47, BA 44/45/46/9, and 39/40)] and; (2) word generation [the phonological information becomes associated with a semantic meaning (BA 30/35/19/27/39) a process that can engage visual imagery (BA 17)] (Karunanayaka et al., 2011). Although these regions were found to be active in the verb generation task from 5 to 18 years old, independent component analysis, and structural equation modeling revealed that the connections between STG and the IFG and within the frontal lobe itself (IFG and MFG) increase and become more left-lateralized from 5 to 18 years. This is in line with the timecourse of continued PFC maturation (Holland et al., 2001; Karunanayaka et al., 2011), and consistent with studies showing increased left-lateralization for verb generation from the age of 5 to 18 years (Holland et al., 2001). Utilizing this same large imaging and behavioral dataset, we can build on the previously published results from this task to explore the relationship between this developing circuitry underlying verb generation and reading skills.

Reading is also a linguistic ability, though unlike the phonological and semantic skills that underlie verbal fluency tasks, it must be explicitly acquired. Since reading is a phylogenetically new skill (roughly, 5000 years old), neuroscientists suggest that the brain uses regions, which are originally devoted to other cognitive functions and utilize them for gaining information from written symbols (see also Price, 2012; Vogel et al., 2013, 2014). Reading demands the translation of abstract graphemes (letters) into their corresponding spoken language sounds (phonemes) that format meaningful words (semantics). It therefore engages visual (cuneus and fusiform gyrus) (McCandliss et al., 2003), auditory/phonological (angular gyrus, STG) (Dehaene-Lambertz et al., 2002), and semantic brain regions (IFG) (Newman and Joanisse, 2011) mainly in the left hemisphere (Chiarello et al., 2009). An efficient synchronization of these processes is needed for fluent reading, which demands the engagement of the executive system (Breznitz, 2006). Behaviorally, reading performance has been suggested to correlate with executive function skills (Christopher et al., 2012; Kieffer et al., 2013; Booth et al., 2014). Many studies have focused on specific aspects of executive functions expected to be related to reading. For example, attention shifting and inhibition (Kieffer et al., 2013), WM, and speed of processing (Christopher et al., 2012), as well as verbal fluency (or verb generation ability), all were found to be correlated with reading ability (Snowling et al., 1997). These findings suggest a major role for executive functions in the reading process.

The neural architecture supporting executive function is described by the dual-networks top-down model (Dosenbach et al., 2008). This model proposed two cognitive control/executive functions networks with different neuroanatomical correlates. The first is the rapid adaptive control network, which is in charge of allocating attention to a cue; uses feedback to affect processing and involves a frontal–parietal circuit. The second network is the set-maintenance network that maintains task goals, which involves a cingulo-opercular circuit. The activation of these networks changes with development due to changes in connectivity between key elements within the network (Dosenbach et al., 2008). Both networks are engaged during reading (Ihnen et al., 2013), but only the functional connectivity between the fronto-parietal network and the fusiform gyrus was found to be positively correlated with reading skill and age (see Vogel et al., 2014).

In this current study, we aim to examine the relations between the maturation of neural networks supporting executive functions and language (during the verb generation task) and reading skill. We focus on the language-network described above (Holland et al., 2007; Karunanayaka et al., 2010, 2011), that includes reading regions related to word recognition (i.e., word form area, BA 37), and regions related to semantic WM (dorsolateral PFC; BA 9, anterior PFC; BA 10), which are part of the executive function network in the dual-network model (Dosenbach et al., 2008). To explore the role of early emerging language networks (Ahmad et al., 2003) in support of later acquired reading skills, we employed a novel longitudinal data analysis model that examines the association between brain activation in the verb generation task before reading skills have become automatic (as early as 7 years old) with reading scores at the age of 17 years. In this longitudinal study, children performed the fMRI verb generation task three times: before reading is fully acquired (7 years old, age range 5–8 years = T1), during acquisition of reading skills (11 years old, age range 9–14 years = T2) and after reading was mastered (17 years old, age range 15–19 years = T3). In typical language development, reading proficiency was found to be affected by the age of reading acquisition, an effect that was found to decrease with age (Zevin and Seidenberg, 2002). Also, a greater variety in strategies used for reading was found in younger individuals as compared to proficient older readers (Waters et al., 1984). We therefore examined the relationship between fMRI activation during verb generation, and reading scores achieved toward adulthood, at the age of 17 years (T3). We hypothesized that the verb generation task would show typical frontal, temporal, and occipital activation, with a trend toward left-lateralized activation with age. We also postulated that reading proficiency would be associated with left-lateralized activation in regions related to executive functions and reading; specifically in the frontal and occipital regions as reading is mastered (i.e., with development) (as was observed in Purcell et al., 2011). Lastly, we hypothesized that at T1 and T2 reading proficiency should be associated with greater activation (during verb generation) of regions in the cingulo-operculum network, because in younger children, maintaining focus on task goals and avoiding errors are of primary importance as reading skills are being acquired. In adolescence, we hypothesized that reading proficiency should be associated with greater activation of the fronto-parietal network at T3 after reading is mastered because in mature, proficient readers, rapid adaptive control mechanisms have been developed and support reading.

## Materials and Methods

### Participants

Sixteen children (eight boys, eight girls) were included in this longitudinal study. Children initially entered the study at the age of 5–8 years and then returned annually for brain imaging and neurocognitive testing. In the present analysis, we focus on imaging data acquired at three time points in the 10-year longitudinal study, when children were of a mean age of 7 ± 0.1.03 (T1), 11.53 ± 1.59 (T2), and 17.18 ± 1.27 (T3) years.

All the participants (*N* = 16) completed the verb generation task during fMRI at T1 and T2. In the last session (T3), only 15 participants completed the scan due to braces in 1 of the participants. However, reading measures were acquired from all 16 individuals at T3.

The study was approved by the Institutional Review Board. Informed consent was obtained from the child’s parent or guardian and assent also obtained from subjects 8 years and older. Exclusion criteria were previous neurological illness, learning disability, head trauma with loss of consciousness, current or past use of psycho-stimulant medication, pregnancy, birth at 37 weeks gestational age or earlier, or abnormal findings at a routine neurological examination performed by an experienced pediatric neurologist. All participants were part of a parent study investigating normal language development in children and were considered “healthy” based on neurological, psychological, and structural measures (Holland et al., 2007; Szaflarski et al., 2012).

All participants were native English speakers. Fifteen were right-handed and one was left-handed according to the Edinburgh Handedness Inventory (Oldfield, 1971). All participants were prescreened for any conditions, which would prevent an MRI scan from being acquired safely. Intelligence was measured using the age appropriate Wechsler intelligence scales upon entry to the study and again 2 and 4 years later as follows: Wechsler Preschool and Primary Scale of Intelligence (WPPSI-R, ages below 6 years) (Wechsler, 1989), Wechsler Intelligence Scale for Children – Third Edition (WISC-III, age 6–16 years) (Wechsler, 1991), and Wechsler Adult Intelligence Scale – Third Edition (WAIS-III, ages 17 years and above) (Wechsler, 1997). Reading was assessed at the third session (T3 at age 17 years) using the Woodcock–Johnson Letter–Word reading test (Woodcock et al., 2001). In this task, the participants were instructed to read as accurately as possible, a list of words written in English, increasing in degree of difficulty. The task was stopped by the administrator after six reading errors in a row.

### fMRI paradigm

The fMRI paradigm consisted of a covert verb generation task as previously detailed (Holland et al., 2001, 2007)] using a 30-s on–off block design. All stimuli were presented using MacStim (White Ant Software, Melbourne, VIC, Australia). Stimuli were presented at a rate of one noun every 5 s, for six stimuli during each 30-s epoch. During the active epochs, the participants were asked to think of appropriate verbs such as “throw” or “kick” to aurally presented concrete nouns such as “ball.” Typically children can think of two or three verbs associated with each noun during each response interval. They are instructed not to move the lips or mouth while covertly generating the verb responses. During the control epochs, participants were asked to bilaterally tap their fingers when they heard a modulated tone. The bilateral finger tapping task was chosen as the control for verb generation since the sensory–motor response to bilateral finger tapping as reported previously (Holland et al., 2001; Szaflarski et al., 2006b). In order to compare the control task to the auditory stimulation and response initiation on the verb generation task, the participants were instructed to sequentially tap the fingers to the thumbs on both hands simultaneously when they hear a tone, in a self-paced manner. Participants were asked to stop tapping after touching each finger to the thumb twice. This control task accomplished five objectives in a pediatric fMRI experiment. First the auditory cue using a tone to pace the finger tapping controls for the auditory stimulation in the verb generation task. Second, tapping fingers provided activation of the motor strip as reference data for each participant as well as a method for examining differences in developmental aspects of BOLD activation from the neurocognitive language paradigm vs. a motor paradigm that should not have very strong developmental influence over the age span tested (Schapiro et al., 2004). It also provided participants with a task to shift their attention away from generating verbs into the control epoch. In addition, paced sequential finger tapping requires motor planning and execution and serves as a control for these processes underlying verb generation task. Finally, this control task is used to provide an indirect measure of participants’ compliance inside the scanner. Yuan et al. (2009) also explored the differences in head motion children during fMRI while performing finger tapping vs. verb generation and did not find a significant difference in motion for these two phases of the task: a key consideration for obtaining high quality fMRI in pediatric subjects. The difficulty level of the fMRI task was selected such that children as young as 5 years old would be readily able to perform the task.

In order to verify that the participants were attentive to the presented nouns during the scan, they were given a yes/no recognition test involving the 25 nouns and 25 distracters. This quiz consisted of a sheet with a list of nouns on it: nouns that were in the task, and foils that were not. Participants were told to mark the words that they remembered hearing during the verb generation task in the scanner.

#### Imaging

An MRI-compatible audio visual system was used for presentation of the stimuli. Details of the techniques used to obtain fMRI data from younger children, as well as the success rates, are given in Byars et al. (2002). EPI–fMRI scan parameters were TR/TE = 3000/38 ms, 125 kHz, FOV = 25.6 cm × 25.6 cm, matrix = 64 × 64, and slice thickness = 5 mm. Twenty-four slices were acquired, covering the entire cerebrum. One hundred ten whole-brain volumes were acquired (the first 10 were discarded during post-processing to insure image contrast at relaxation equilibrium) for a total scan time of 5 min 30 s. Techniques detailed elsewhere (Byars et al., 2002) were used to acclimatize the participants to the MRI procedure and render them comfortable inside the scanner. Soft head restraints were used to minimize head motion. In addition to the fMRI scans, whole-brain T1 weighted MP-RAGE scans were acquired for anatomical co-registration. All imaging was performed using a 3 T, head only MRI scanner (Bruker Medspec 30/60).

### Data analysis

Data were analyzed using Cincinnati Children’s Hospital Image Processing Software (CCHIPS), written in IDL (Research Systems Inc., Boulder, CO, USA). Image data were corrected for Nyquist ghosts and geometric distortion using multi-echo reference method (Schmithorst et al., 2001), and was motion-corrected using pyramid co-registration (Thevenaz et al., 1998): we performed three-dimensional ridged body transformation to align the volumes. This resulted in six motion parameters. These parameters were included as regressors in the first-level general linear model (GLM) analysis for each subject’s data. To ensure that the level of motion did not differ across the three sessions, the mean motion was reported as well as included in an analysis of variance (ANOVA). We also used a within group *t*-test analysis to compare the motion levels between the task (when generating verbs) and the contrast conditions (when tapping fingers). In addition, time points with excessive motion were rejected from the post-processing pipeline. We used a mutual information cost function for rejecting motion corrupted frames of fMRI data as previously described for this same cohort of longitudinal subjects (Szaflarski et al., 2006). As mentioned in our previous studies, covert and overt verb generation resulted in the same head motion in this same cohort of children (Vannest et al., 2009). Although the data included in the current analysis are from a slightly different block-periodic version of the verb generation task, we did not find differences in head motion during the covert verb generation and finger tapping conditions (Yuan et al., 2009). All data met the criterion of median voxel displacement in the center of the brain <2 mm. The fMRI data were transformed into stereotaxic space (Talairach and Tournoux, 1988) using a linear affine transformation (see Muzik et al., 2000). The use of the Talairach standard for children aged 5 years and above has been shown to produce minimal errors in co-registration for group analysis. Consequently, we are confident that using the adult Talairach standard in the present study will not substantially alter the findings of the current study (Wilke et al., 2002; Altaye et al., 2008). Note that the imaging data for this study were acquired over a period of 12 years and that the methods for fMRI analysis have improved considerably over this period of time. After data acquisition on all participants was completed, 12 years after the longitudinal study began we processed all of the data from all participants specifically for this report using the analysis pipeline as described above.

### Group activation map

A GLM and random-effects analysis were used in order to detect the group activation for T1, T2, and T3 children for the contrast between conditions of verb generation > finger tapping contrast. Images of the *t*-maps generated by this first-level contrast were thresholded for visual inspection purposes to *p* < 0.001, corrected via Monte Carlo simulation using a cluster of 30 voxels (Forman et al., 1995).

#### Regression

We used regression in second level analyses in order to examine the association between reading scores from the “Letter–Word” subtest (from the Woodcock–Johnson battery) from the third session (T3) and fMRI activation during the verb generation task at T1–T3. Following the first-level computation of individual *t*-score maps for the verb generation > finger tapping contrast, whole-brain voxel-wise group maps, were computed using Spearman’s rank-correlation coefficient. These maps were based on unthresholded *t*-maps from the first-level analysis and each subject’s behavioral score included as a predictor of activation (e.g., “Letter–Word” subtest from the Woodcock–Johnson III). Images were thresholded to *p* < 0.001, uncorrected. Cluster size for each group was 35 voxels.

#### Lateralization index calculation

A lateralization index (LI) was also calculated for each subject in the three age groups based on the unthresholded individual *z*-score maps. The LI represents a relative hemispheric difference for an individual that is self-normalizing in terms of relative BOLD activity. Since this index is non-dimensional, it provides a convenient means of comparing groups of subjects across different scanning sessions. As noted later, the brain areas included for LI calculation were determined so that they were consistent across the groups while still conforming to the traditional standard of regions of interest (ROI) selection for language lateralization. Every effort was made to insure that the methodologies applied in the LI calculation of the three subject groups were compatible.

Several ROIs were chosen for the purpose of the current analysis using an LI toolbox implemented in CCHIPS. Language-network regions were defined for LI calculation based on the composite map of activation obtained from the fMRI verb generation task from all subjects at T3. First, we chose a frontal ROI corresponding to the traditional “Broca’s area”; we retained all active voxels within and contiguous to this area, so the frontal ROI included the entire IFG (BA 44, 45, 47) and extensions into additional dorsolateral prefrontal regions, including portions of the MFG (BA 46, 48, 49) and precentral gyrus (BA 46). We also selected a temporal language ROI based on the traditional “Wernicke’s area,” defined functionally by the group activation map. This ROI extended from the temporal plane (BA 41, 42) inferiorly through the middle temporal gyrus (MTG) to the margin of the ITG (including portions of BA 22, 21, 37) but did not include posterior aspects (BA 22, 39). ROIs for the right hemisphere homologs were established by reflecting the coordinates to the right hemisphere.

Using the same approach, we defined ROIs for reading (fusiform gyrus, BA 37) and for executive functions (dorsolateral PFC, BA 9; and the anterior PFC, BA 10) (after Purcell et al., 2011). We retained all active voxels within and contiguous to these areas and defined them as two additional separate ROIs on the left and for the right hemisphere homologs based on the correlation map of activation obtained from the fMRI verb generation task correlated with the WJ-III reading scores from all subjects at T3.

Lateralization indexes for language-network regions were computed both for the composite *t*-maps and for the regression maps; the LIs for the reading and executive functions-related regions were then computed separately from the regression maps. Only voxels with *z*-scores greater than or equal to the mean *z*-score within an ROI for each individual subject were used in the calculation of LIs. Voxels above this mean *z*-score threshold were counted and an LI was defined as the difference in the number of activated voxels, summed independently for the left and right ROIs, divided by the sum total of active voxels in the left plus right ROI. According to this formula, a positive LI indicated left hemisphere lateralization and a negative number indicated right lateralization. These LI values were used to construct the lateralization growth curves (a linear regression line).

## Results

### Neuropsychological testing

Neuropsychological testing results verified that the participants had normal to above average scores in reading and verbal abilities. Mean Wechsler verbal and non-verbal IQ remained stable during development. At T1: verbal scaled IQ = 115.9 ± 9.23 (range = 104–139), non-verbal scaled IQ = 112.9 ± 12.23 (range = 96–130), at T3: verbal scaled IQ = 109.75 ± 0.37 (range = 99–139), non-verbal scaled IQ = 114.91 ± 14.71 (range = 95–123).

### Verb generation – post-scanning measurement

The average number of correct responses for the verb generation post-test outside the scanner at T2 and T3 were 12.4/25 ± 3.6 and 15.05/25 ± 2.4, respectively. Range of possible scores is 0–25 correct choices.

### Random-effects analysis

**Figure 1:**
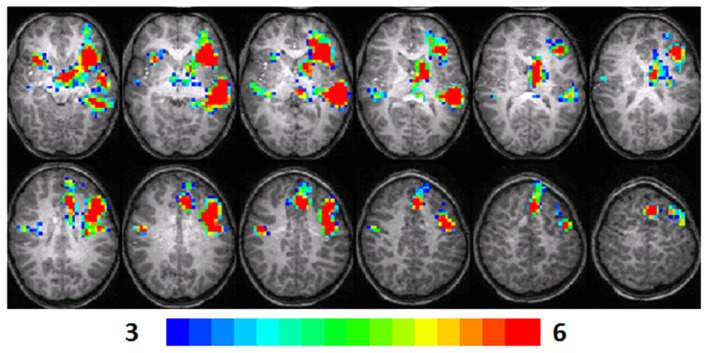
**Composite fMRI activation maps for the verb generation (verb generation > finger tapping) in T1 children (*N* = 16)**. The contrast is significant at *p* < 0.001 (corrected), slice thickness is 5 mm for these contiguous slices. Slices range from *z* = 13 to 24 in the Talairach frame. Cluster size is 30 voxels. Higher significance is indicated in hotter color (*t* threshold ranged from 3 to 6, see scale in the bottom of the figure). Figure is presented in a radiological orientation (L = R, R = L).

**Figure 2:**
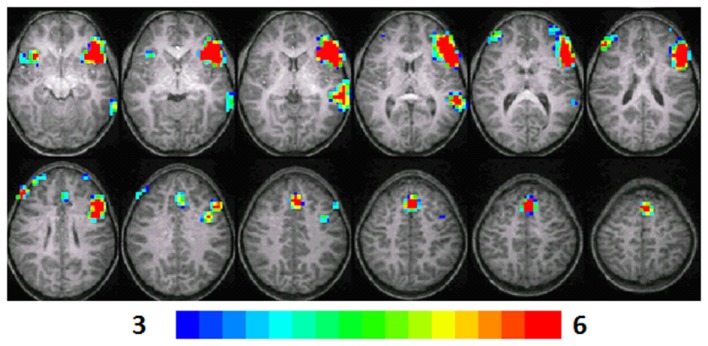
**Composite fMRI activation maps for the verb generation (verb generation > finger tapping) in T2 children (*N* = 16)**. The contrast is significant at *p* < 0.001 (corrected), slice thickness is 5 mm for these contiguous slices. Slices range from *z* = 13 to 24 in the Talairach frame. Cluster size is 30 voxels. Higher significance is indicated in hotter color (*t* threshold ranged from 3 to 6). Figure is presented in a radiological orientation (L = R, R = L).

**Figure 3:**
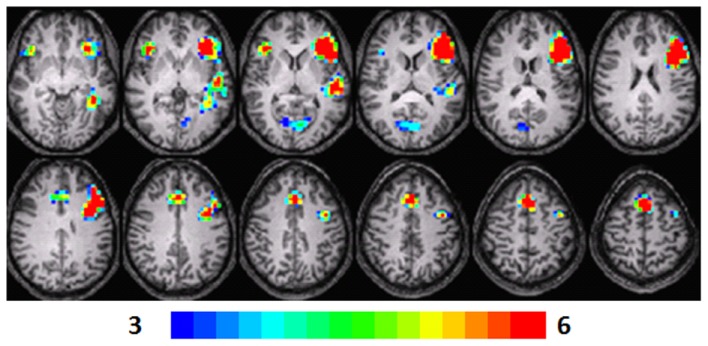
**Composite fMRI activation maps for the verb generation (verb generation > finger tapping) in T3 children (*N* = 15)**. The contrast is significant at *p* < 0.001 (corrected), slice thickness is 5 mm for these contiguous slices. Slices range from *z* = 13 to 24 in the Talairach frame. Cluster size is 30 voxels. Higher significance is indicated in hotter color (*t* threshold ranged from 3 to 6). Figure is presented in a radiological orientation (L = R, R = L).

The statistical parameter maps for verb generation > finger tapping for T1–T3 in the longitudinal cohort shown in Figures 1–3 are consistent with previous studies using this task (Holland et al., 2007; Szaflarski et al., 2012). While there are many areas of activation that are common across ages in the cohort, there are also variations.

#### T1 children

Significant activation was found in the left and right insula (BA 13) and left IFG (BA 13, 9), left fusiform gyrus (BA 20), left STG (BA 22), left MTG (BA 21), left superior frontal gyrus (SFG) (BA 6), left cingulate gyrus (BA 32), left thalamus, right precentral gyrus (BA 6), and left and right parahippocampal gyrus. Talairach coordinates of cluster centroids are listed in Table 1 (see Figure 1).

**Table 1:** **Group composite contrast (verb generation > finger tapping) in T1, T2, and T3 participants**.

Region	BA	Cluster size	Cluster centroid		
			*X*	*Y*	*Z*
**T1 CHILDREN**					
Left insula	13	133	−30	22	0
Left inferior frontal gyrus	13	66	−34	27	14
	9	150	−39	8	29
Left superior temporal gurus	22	155	−51	−24	3
Left middle temporal gyrus (sub-cluster)	21	26	−48	−36	−5
Left fusiform gyrus (sub-cluster)	20	22	−44	−37	−10
Left superior frontal gyrus	6	32	−8	15	57
Left parahippocampal gyrus		202	−26	2	−15
left thalamus		142	−8	15	57
Left cingulate gyrus	32	64	−7	22	39
Right precentral gyrus (frontal lobe)	6	41	42	−7	30
Right parahippocampal gyrus		224	26	4	−18
Right insula	13	48	42	−18	13
**T2 CHILDREN**					
Left middle frontal gyrus	46	15	−38	46	15
Left middle frontal gyrus (sub-cluster)	9	63	−43	12	27
Left inferior frontal gyrus (sub-cluster)	47	88	−40	18	−13
	13	345	−44	23	7
Left middle temporal gyrus	22	94	−60	−35	5
Left middle temporal gyrus (sub-cluster)	21	21	−58	−35	−13
Left anterior cingulate	32	91	−2	19	40
Right inferior frontal gyrus	13	69	38	13	−13
Right middle frontal gyrus	10	52	40	38	22
**T3 CHILDREN**					
Left parahippocampal gyrus (limbic lobe)	36	82	−39	−37	−6
Left superior temporal gyrus	22	87	−47	−22	4
Left inferior frontal gyrus	13	284	−38	24	8
Left middle frontal gyrus (sub-cluster)	9	78	−38	14	26
	6	28	−37	4	40
Right middle frontal gyrus	8	158	0	19	43
Right cuneus	30	56	1	−68	7
Right Inferior frontal gyrus	47	44	37	23	1

#### T2 children

Significant activation was observed in the left and right MFG (BA 46, 9, 10), left IFG (BA 47), left MTG (BA 21, 22), right IFG (BA 13), and left anterior cingulate (BA 32). Talairach coordinates of cluster centroids are listed in Table 1 (see Figure 2).

#### T3 children

Significant activation was observed in the left STG (BA 22), left MFG (BA 46, 9, 8), left parahippocampal gyrus (BA 36), left (BA 13) and right IFG (BA 47), and right cuneus (BA 30). Talairach coordinates of cluster centroids are listed in Table 1 (see Figure 3).

### Regression analysis maps

Regression analyses maps using the “Letter–Word” standard scores from the Woodcock–Johnson III at T3 as a predictor, revealed significant positive correlations with the contrast of verb generation > finger tapping in the children at three different age points.

#### T1 children

**Figure 4:**
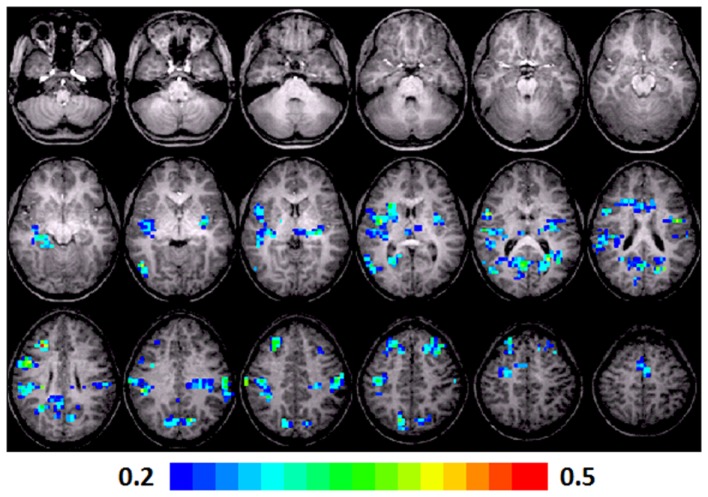
**Regression maps showing a positive correlation for activation during verb generation scans in T1 with the Letter–Word score from the Woodcock–Johnson III (*N* = 16) in T3**. All activated pixels meet significance threshold of *p* < 0.05, corrected. Slices range from *z* = 7 to 24 in the Talairach frame. Cluster size is 35 voxels. Higher significance is indicated in hotter color (*r* value ranged from 0.2 to 0.5, see scale in the bottom of the figure). Figure is presented in a radiological orientation (L = R, R = L).

A significant positive correlation was observed in the left insula (BA 13), left and right MFG (BA 8, 6), left posterior cingulate (BA 30), left anterior cingulate (BA 24), left thalamus, right precentral gyrus (BA 44), right superior temporal lobe (BA 41), right MTG (BA 37), right cuneus (BA 7), and right posterior cingulate (BA 23, 30). Talairach coordinates of cluster centroids are listed in Table 2 (see Figure 4).

**Table 2:** **Regression analysis of MRI data from the verb generation task at T1, T2, and T3 participants with Letter–Word reading standard scores at T3**.

Region	BA	Cluster size	Cluster centroid		
			*X*	*Y*	*Z*
**T1 CHILDREN**					
Left posterior cingulate	30	32	−19	−58	17
Left insula	13	25	−41	−20	26
Left middle frontal gyrus (frontal lobe)	8	33	−27	20	42
Left thalamus		41	−9	−20	7
Left anterior cingulate cortex	24	35	−1	15	20
Right middle frontal gyrus (frontal lobe)	6	37	7	−4	56
Right precentral gyrus (frontal lobe) (sub-cluster)	44	20	46	5	10
Right inferior frontal gyrus (frontal lobe) (sub-cluster)	9	17	47	3	26
Right posterior cingulate (limbic system)	23	9	7	−51	25
Right posterior cingulate (sub-cluster)		55	10	−60	17
Right posterior cingulate (sub-cluster)	30	14	20	−54	10
Right transverse temporal gyrus (A1)	41	72	44	−25	12
Right superior temporal gyrus (sub-cluster)	41	9	46	−38	15
Right middle temporal gyrus	37	57	49	−58	5
Right cuneus (occipital lobe)	7	51	9	−66	30
**T2 CHILDREN**					
Left fusiform gyrus (temporal lobe)	37	48	−43	−50	−10
Left superior temporal gyrus (sub-cluster)	41	20	−38	−42	5
Left anterior cingulate	32	47	−5	31	20
Left middle frontal gyrus	10	36	−9	50	5
Left fusiform gyrus (occipital lobe)	19	40	−38	−73	−12
Right caudate (temporal lobe)		25	36	−30	−5
Right culmen (anterior) (sub-cluster)		25	25	−29	−25
Right hypothalamus		37	1	−2	−10
Right hippocampus (temporal lobe) (sub-cluster)		17	34	−29	−10
Right fusiform gyrus (occipital lobe)	19	35	34	−77	−12
**T3 YEAR OLD CHILDREN**					
Left superior temporal gyrus (temporal lobe)	38	44	−39	14	−22
Left fusiform gyrus (temporal Lobe)	20	96	−36	−38	−15
Left parahippocampal gyrus (limbic lobe)	34	41	−16	−7	−18
Left superior temporal gyrus	41	111	−44	−37	4
Left insula	13	100	−37	−20	9
Left insula (sub-cluster)	13	44	−35	−13	14
Left middle frontal gyrus (frontal lobe)	10	86	−37	37	9
Left middle frontal gyrus	9	97	−33	15	27
Left cuneus (occipital lobe)	18	58	−6	−76	23
Left precuneus (parietal lobe, sub-cluster)	7	16	−19	−55	56
Right medial frontal gyrus (frontal lobe)	6	81	6	−19	53
Right precuneus	31	76	18	−74	28
Right posterior cingulate	30	75	31	−74	12
Right lingual gyrus (occipital lobe)	18	72	17	−54	5

#### T2 children

**Figure 5:**
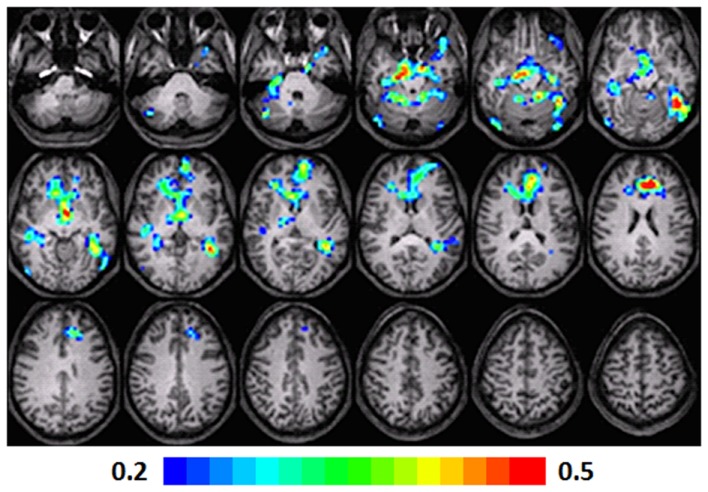
**Regression maps showing a positive correlation for activation during verb generation scans at T2 and the Letter–Word score from the Woodcock–Johnson III (*N* = 16) from T3**. All activated pixels meet significance threshold of *p* < 001 uncorrected. Slices range from *z* = 7 to 24 in the Talairach frame. Cluster size is 35 voxels. Higher significance is indicated in hotter color (*r* value ranged from 0.2 to 0.5). Figure is presented in a radiological orientation (L = R, R = L).

A significant positive correlation was observed in left and right fusiform gyri (BA 37, 19), left STG (BA 41), left anterior cingulate (BA 32), and the MFG (BA 10). In the right side, a significant correlation was observed in the caudate, hypothalamus, and hippocampus. Talairach coordinates of cluster centroids are listed in Table 2 (see Figure 5).

#### T3 children

**Figure 6:**
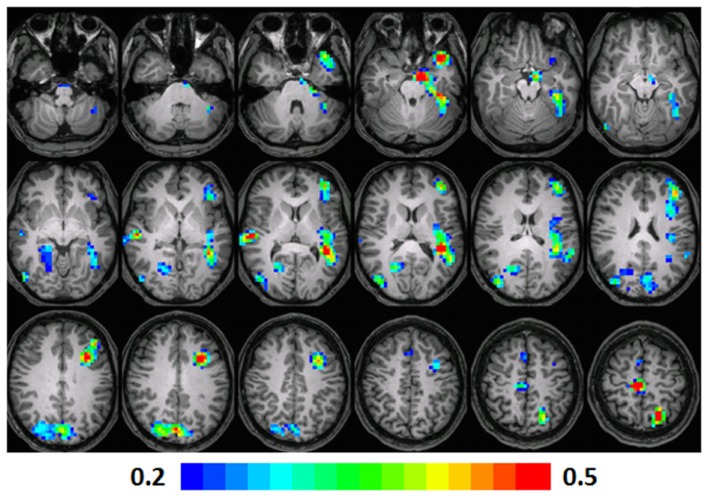
**Regression maps showing a positive correlation for activation during verb generation scans from T3 and the Letter–Word score from the Woodcock–Johnson III (*N* = 15) at T3**. The activated pixels meet significance at *p* < 0.001, uncorrected, higher significance is indicated by hotter color. Slices range from *z* = 7 to 24 in the Talairach frame. Cluster size is 35 voxels. Higher significance is indicated in hotter color (*r* value ranged from 0.2 to 0.5). Figure is presented in a radiological orientation (L = R, R = L).

A significant positive correlation was observed in the left STG (BA 41, 38), left MFG (BA 9, 10), left insula (BA 13), left fusiform gyrus (BA 20), left parahippocampal gyrus (BA 34), and the left precuneus (BA 7) and cuneus (BA 18). A significant correlation was also observed in the right MFG (BA 6), precuneus (BA 31), posterior cingulate (BA 30), and lingual gyrus (BA 18). Talairach coordinates of cluster centroids are listed in Table 2 (see Figure 6).

### Age-related changes in lateralization

**Figure 7:**
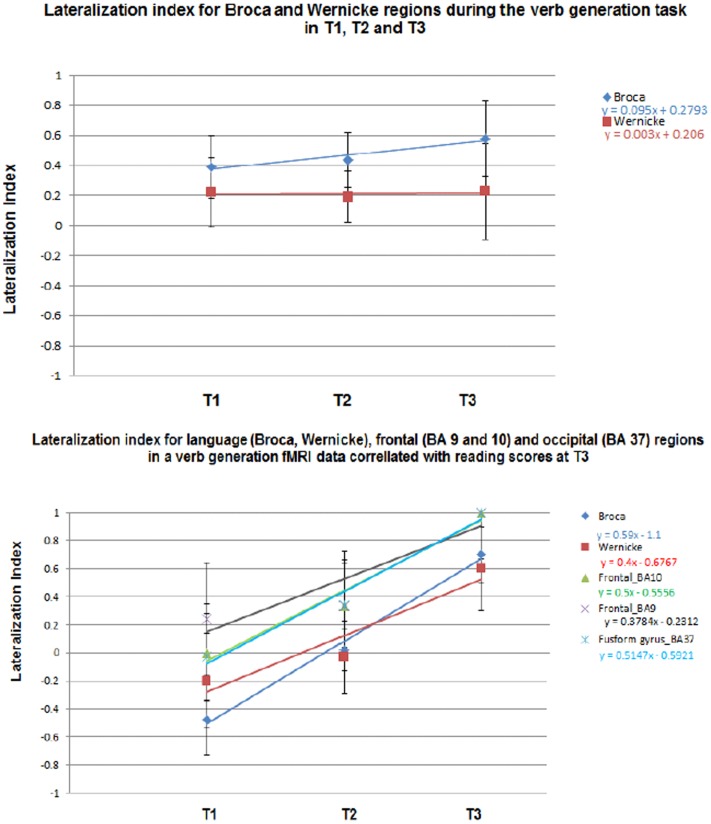
**Lateralization index (LI) for the composite (upper) and the regression maps (lower)**. LI was calculated on language-related regions (Broca and Wernicke) in the composite maps in T1–T3. LI was also calculated on language (Broca and Wernicke), executive functions (dorsolateral prefrontal cortex, anterior prefrontal cortex), and reading regions (fusiform gyrus). Standard deviations representing the distribution of individual LIs are indicated.

In Figure 7 the LI for each group is plotted as a function of age (for T1, T2, and T3) for frontal and temporal language-network ROIs illustrated in the composite maps in Figures 1–3. A significant age-related increased left-lateralization was found for the frontal ROI (i.e., “Broca”) (*R*^2^ = 0.92). Although we observed a leftward lateralization trend in the temporal region as well (i.e., “Wernicke”), age-related change in this posterior ROI was more modest (*R*^2^ = 0.02). Results suggest that age accounted for as much as 92% of individual variance in LI in the activation in Broca and for 2% of individual variance *N* LI in the activation in Wernicke. See upper part of Figure 7.

An examination of the language ROIs from the regression maps with reading scores in Figures 4–6, revealed increased left-lateralization along development as shown in the lower panel of Figure 7 for the frontal (green triangles, *R*^2^ = 0.99) and temporal (red squares, *R*^2^ = 0.9) language-network ROIs. Greater age-related changes in lateralization, were found for the reading regions as well (turquoise X, fusiform gyrus; *R*^2^ = 0.97), and for the executive functions regions (purple X, BA 9: *R*^2^ = 0.83, BA 10: *R*^2^ = 0.96). See lower part of Figure 7. Results suggest that age and reading proficiency accounted for 99% of individual variance in LI in the activation in Broca and for 90% of individual variance in LI in the activation in Wernicke. It also emphasized that age and reading proficiency accounted for 97% of individual variance in LI in the activation in the Fusiform gyrus and for 83 and 96% of individual variance in LI in the activation in executive functions regions (BA 9 and 10, respectively). These findings indicate that those regions positively correlated with reading scores became more left-lateralized with age. This was true for functionally defined regions of significant correlation for reading scores and brain activation as well as for regions that were active in the first level analysis in Broca and Wernicke’s areas. The fact that this lateralization trend is similar for all of these regions (slope of LI vs. time in the range of 0.37–0.59) suggests that these regions are all connected in support of reading and language skill and are developing at a similar pace, even though the regression coefficient is not significant for the primary regions.

## Discussion

In the current study, we aim to examine the relationship between reading proficiency and the maturation of neural networks related to executive functions and language, using the verb generation task. The verb generation task draws on language and executive skills that improve during development (Missier and Crescentini, 2011) and correlate with reading ability (Snowling et al., 1997); thus, we were able to study participants in the youngest group (T1) who entered the study before they formally acquired reading skills or before reading was fully acquired, and examine activation in regions supporting language and executive skills without requiring a reading task.

In line with our hypothesis, our results demonstrate a trend of more left-lateralized activation during the verb generation task from T1 to T3. In other words, age was found to account for the increasing left LI in language-related regions. Specifically, we observed increasingly leftward lateralization of the inferior/middle frontal region with development, as reported previously in a super-set including the participants of the current analysis (Holland et al., 2001, 2007). This trend was less robust in the temporal language region, as previously observed, due to a weaker reliance on auditory–phonological-related regions during this task (Holland et al., 2007). A similar trend of left-lateralized activation with development was observed by Gaillard et al. (2000) while administering the verb generation task to participants age 10.7 and 28.7 years. This developmental shift to the left hemisphere for linguistic tasks was also reported in other studies (Szaflarski et al., 2006a) and reflects the specialization of the left hemisphere for language. These results are not confounded by differences in motion across age or between the different task conditions (i.e., generating verbs vs. tapping fingers). Although the head motion data were regressed out of the analysis, the absence of the effect of age on motion level, confirms that these findings of increased left-lateralization with age are not because of motion differences (see also Gaillard et al., 2003).

The main goal of the current study was to identify the developmental changes in the activation of the neural circuitry supporting language and executive functions (during the verb generation task in T1, T2, and T3) corresponding to proficient reading at T3. Our results confirm our second hypothesis: reading proficiency was found to be associated with left-lateralized activation in regions related to language and profound left-lateralization in executive functions (BA 9, 10) and reading (BA 37). In general, greater reading scores in T1 were associated with a bilateral and more widespread brain activity, in addition to the “classical” reading regions. This involved the right STG and MTG, which are related to phonology and bilateral activation of production and semantic-related regions such as the insula. These findings extend our previous results (Holland et al., 2001, 2007) by showing that age and reading proficiency account for increasing left-lateralization in regions related to language, reading, and executive functions. This is in line with findings suggesting lesser reliance on phonological and semantic processes and greater reliance on orthographic processes during reading proficiency (Ehri, 2005), and neuroimaging findings of greater left-lateralization during reading along development (Turkeltaub et al., 2003). With age, the association of greater reading scores and brain activation in the verb generation task was found to be more specific to executive functions and reading-related regions. At T2 and T3, greater reading scores were associated with more leftward LI for language-related regions (frontal and temporal) than at T1, but even more leftward LI scores for frontal and occipital regions related to executive functions and reading, respectively. Since better reading is associated with greater reliance on word recognition (i.e., “orthographic route”) and less on decoding (i.e., “phonological route”), these results might demonstrate the decreased role of language regions (i.e., phonology–auditory route) and the increased role of the orthography–visual route in reading development (for more information see Coltheart et al., 2001; dual route model).

Positive correlation of word reading scores and the activation of the fusiform gyrus was observed only in T2 and T3, whereas children in the T1 group showed greater right cuneus and left precuneus activation. We suggest that because younger age children have not reached reading proficiency and the mental lexicon has not fully been established, there is heavier load on regions related to phonological processing (e.g., STG, MTG) and less on regions related specifically to automatic word recognition (e.g., fusiform gyrus) earlier in development. At T2, children are using orthographic processing regions as they become more proficient readers (Ehri, 2005) and therefore show a correlation between reading proficiency with bilateral fusiform gyri activation. Finally, left sided fusiform gyrus activation is observed at T3, as expected from proficient and automatic readers (van der Mark et al., 2011). The activation of the fusiform gyrus, lingual gyrus, and other occipital regions may be related to visual imagery during the verb generation task (see also Karunanayaka et al., 2011). This process was also found to correspond to greater reading proficiency (Horowitz-Kraus et al., 2013).

Note that the ANOVA we performed to assess the effect of age on motion did not reveal significant age differences [*F*(3,45) = 0.48, *p* > 0.05]. Namely, the level of motion did not differ across the age points. No significant differences were found within groups either, suggesting that motion level for the task and contrast conditions was relatively equal (see Table 3).

**Table 3:** **Motion measures, mean, and standard deviation (SD) for children at T1, T2, and T3**.

	Mean (SD) (total: generating verbs and tapping fingers)	Mean (SD) (generating verbs)	Mean (SD) (tapping fingers)	*t* Test (generating verbs vs. tapping fingers) (ns)
T1 children	0.3 (0.22)	0.33 (0.22)	0.3 (0.21)	0.38
T2 children	0.39 (0.36)	0.43 (0.53)	0.37 (0.22)	0.26
T3 children	0.33 (0.3)	0.32 (0.31)	0.34 (0.3)	0.43

*Means and standard deviations (SDs) for motion measures for the total task (generating verbs and finger tapping), the task condition (generating verbs), and for the contrast condition (finger tapping) across the different age groups. No significant differences were found between the different age groups for the total motion parameter as well as between the task and contrast conditions (left column)*.

The frontal lobe matures with age, along with concomitant improvements in executive functions (Segalowitz and Davies, 2004). Giedd observed that gray and white matter maturation, which includes completion of myelination and synaptic pruning, in the parietal and frontal regions peaks at 16 years old, so the cognitive abilities centered in these regions are mature as well (Giedd et al., 1999). Some later findings point at an inverse relationship between cortical gray matter density reduction and brain growth primarily in the superior frontal regions that control executive functioning even in 30-year-old adults (Sowell et al., 2001). Executive functions, which support inhibition, WM, planning, and attention, likely develop throughout adolescence together with the maturation of the frontal and parietal cortices (Casey et al., 2000; Luna et al., 2010).

Since our sample is children in the age range of 7–17 years, frontal lobe maturation is still in progress, and verb generation may demand a reliance on different neural-circuits related to executive functions for T1 and T2 children as compared to T3. We hypothesized that the fronto-parietal network may be more engaged in older children, and our finding of greater left frontal and parietal (precuneus) activation correlated with reading proficiency at T3 supports this hypothesis. As suggested by the dual-networks model, the fronto-parietal network may be in charge of rapid adaptive control and attention allocation, which are part of executive functions (Dosenbach et al., 2008). This network has been previously found to correlate with higher reading scores (Vogel et al., 2014). As adolescents become proficient readers, rapid monitoring is required to process the written stimuli in order to achieve fluent reading. This same rapid control mechanism that contributes to fluent reading is also engaged to a greater degree during verb generation in those participants who are the most proficient readers. The cingulo-opercular network, supporting maintenance of task goals and error monitoring, is also observed, in part, at T3: the insula was also positively correlated with higher reading scores.

We also found that the cingulo-opercular network was more engaged in younger children; there was activation of the insula at T1 and of the anterior cingulate cortex at T1 and T2. These results suggest that greater task maintenance and error monitoring is needed by younger children – as they perform the verb generation task, semantic associations among words are not as well-established, and selecting appropriate responses involves greater resources. This is consistent with a previous finding from the verb generation task when nouns presented to the participants involved greater conflict and the need for more attentional resources to resolve this conflict (Barch et al., 2000).

The activation of the anterior cingulate cortex in T1 and T2 was also positively correlated with higher reading scores at the age of 17 years (T3). This suggests that children who go on to become more proficient readers engage this region to a greater degree even early in development. Before reading becomes automatic, there are several competing representations of new words as they are encountered, until the orthographic route becomes stable (Coltheart et al., 2001; dual route model). Young children who devote increased resources to resolving these conflicting representations may ultimately become better readers, suggesting that, more generally, executive functions play a role in the reading acquisition process. We suggest that by adolescence, as linguistic and executive abilities increase, conflict may decrease, resulting in the absence of activation in the anterior cingulate cortex in the verb generation task at T3. The anterior cingulate cortex also supports error detection and is active when making reading errors (Horowitz-Kraus and Breznitz, 2008), and the absence of association between reading ability and the activation in this region at T3 years might be due to less error detection activation during the verb generation task as well as in reading and less need for set-maintenance during these processes, which become automatic. Vogel et al. (2013) found no developmental changes in fronto-parietal and cingulo-opercular networks when examining these networks using resting-state functional connectivity. Our regression maps as well as the LI values from the frontal ROIs suggest that while these executive functions networks remain equally active across development, when examining a specific task; one network may be more active than another at different developmental stages.

To conclude, our results support the utilization of language and executive functions regions for development of proficient reading. These domains also have a differential role of executive functions in reading development: greater role of the set-maintenance network before reading becomes automatic (T1 and T2) and greater activation of the rapid, adaptive control network when reading becomes proficient. Our results strengthen previous studies, which pointed at the crucial role of executive functions in the reading process, highlighting the differential need in sub-components of executive functions along development. Clinically, the results provide neuro-functional support for the importance of intact executive functions in future reading development, by means of early diagnosis and intervention, especially among populations with reading impairments (dyslexia, attention deficit hyperactive disorder). These conclusions should be taken in the context of the current study’s limitations: (1) We did not acquire fMRI data during a reading task, so we cannot compare the regression maps with reading scores and verb generation to actual activation during reading in the same participants; (2) there is a reading exposure at 7 years old (and especially since the age range of this group was relatively wide) so the youngest group of participants is not completely “naïve” to reading. Adding to this ambiguity in the sample is the lack of assessment of reading ability at the younger age points. An additional study enrolling children younger than 7 years old should be done to verify whether the same phenomenon exists also in a younger age. (3) We did not assess the verb generation ability after the scan at 7 years old since the children were too young. (4) The only reading measure that was used in the current study was a word reading task. Although word reading is highly related to reading comprehension, these two abilities rely on different basic cognitive abilities (see Christopher et al., 2012 for more information) as well as different white matter tracts (Horowitz-Kraus et al., 2013). Also, whereas an untimed word reading task, as was used in the current study involves phonological and orthographical processing, a timed word recognition task relies additionally on speed of processing (see Wolf and Bowers, 1999; Hart et al., 2010). Therefore, a correlation of the fMRI data from the verb generation task with a timed word reading task may result in less activation of phonological-related regions (e.g., STG). A future study should look at the longitudinal difference in the fMRI verb generation correlates with these two measures. Despite these limitations, it is important to note that there are only a few studies assessing the relationship between executive functioning and reading and the current study is the first to do so longitudinally. Therefore, our findings (regional correlation of later reading abilities with early fMRI results) offer the possibility of a novel predictive biomarker for future reading ability.

## Conflict of Interest Statement

The authors declare that the research was conducted in the absence of any commercial or financial relationships that could be construed as a potential conflict of interest.
